# LightCF-Net: A Lightweight Long-Range Context Fusion Network for Real-Time Polyp Segmentation

**DOI:** 10.3390/bioengineering11060545

**Published:** 2024-05-27

**Authors:** Zhanlin Ji, Xiaoyu Li, Jianuo Liu, Rui Chen, Qinping Liao, Tao Lyu, Li Zhao

**Affiliations:** 1 Hebei Key Laboratory of Industrial Intelligent Perception, North China University of Science and Technology, Tangshan 063210, China; zhanlin.ji@ncst.edu.cn (Z.J.); lixiaoyu@stu.ncst.edu.cn (X.L.); ljn6782022@163.com (J.L.); 2 College of Mathematics and Computer Science, Zhejiang A&F University, Hangzhou 311300, China; 3 Beijing Tsinghua Changgung Hospital, School of Clinical Medicine, Institute for Precision Medicine, Tsinghua University, Beijing 100084, China; cra01052@btch.edu.cn (R.C.); lqpa00594@btch.edu.cn (Q.L.); 4 Beijing National Research Center for Information Science and Technology, Institute for Precision Medicine, Tsinghua University, Beijing 100084, China

**Keywords:** polyp segmentation, large kernel attention, visual attention mamba, PSA

## Abstract

Automatically segmenting polyps from colonoscopy videos is crucial for developing computer-assisted diagnostic systems for colorectal cancer. Existing automatic polyp segmentation methods often struggle to fulfill the real-time demands of clinical applications due to their substantial parameter count and computational load, especially those based on Transformer architectures. To tackle these challenges, a novel lightweight long-range context fusion network, named LightCF-Net, is proposed in this paper. This network attempts to model long-range spatial dependencies while maintaining real-time performance, to better distinguish polyps from background noise and thus improve segmentation accuracy. A novel Fusion Attention Encoder (FAEncoder) is designed in the proposed network, which integrates Large Kernel Attention (LKA) and channel attention mechanisms to extract deep representational features of polyps and unearth long-range dependencies. Furthermore, a newly designed Visual Attention Mamba module (VAM) is added to the skip connections, modeling long-range context dependencies in the encoder-extracted features and reducing background noise interference through the attention mechanism. Finally, a Pyramid Split Attention module (PSA) is used in the bottleneck layer to extract richer multi-scale contextual features. The proposed method was thoroughly evaluated on four renowned polyp segmentation datasets: Kvasir-SEG, CVC-ClinicDB, BKAI-IGH, and ETIS. Experimental findings demonstrate that the proposed method delivers higher segmentation accuracy in less time, consistently outperforming the most advanced lightweight polyp segmentation networks.

## 1. Introduction

Colorectal cancer (CRC) ranks as one of the most prevalent malignant tumors globally, with its incidence steadily rising. By 2024, it is expected to become the leading cause of cancer-related deaths among men under 50 in the United States and the second leading cause among women of the same age group [1]. Fortunately, early screening significantly lowers both the incidence and mortality rates of colorectal cancer, boasting a five-year survival rate of approximately 90% for early-stage patients [2]. Moreover, studies show that almost 95% of colorectal cancers develop from tumorous polyps on the walls of the colon or rectum [3]. Therefore, the early detection and excision of tumorous polyps are vital for the successful treatment of colorectal cancer. Colonoscopy is considered the most accurate and widely used screening method for colorectal cancer at present.

Colonoscopy can reduce the risk of death from colorectal cancer by 68% [4]. However, the precision of colonoscopy significantly relies on the physician’s expertise and environmental factors. On one hand, the detection and treatment of polyps in colonoscopy videos rely entirely on the doctor’s experience, where prolonged focus and a high level of mental state also affect the doctor’s operational level and diagnostic rate [5]. On the other hand, due to the complex structure of polyps and inconsistency in image quality, there is some subjective variation among different doctors in their observation and judgment during colonoscopy, leading to inconsistent diagnostic results [6]. Moreover, studies have shown that about 25% of tumorous polyps are missed in routine colonoscopy examinations [7]. Therefore, it is crucial to develop an accurate and efficient method to assist doctors in diagnosing colorectal polyps in clinical practice.

However, due to the polyps’ varying sizes, colors, and shapes, developing a segmentation network that suits all types of polyps and consistently achieves satisfactory performance is very difficult. Additionally, complex environmental factors in colonoscopy videos, such as inadequate lighting, mucus, or foam residues, make it difficult for the network to effectively extract discriminative features of polyps and accurately demarcate the fine boundaries between polyps and the surrounding mucosal tissue. Moreover, in clinical settings, segmentation algorithms need to have high real-time capability so that physicians can promptly diagnose and treat. Nevertheless, in complex contextual situations, it is extremely difficult to achieve satisfactory segmentation outcomes while maintaining real-time performance. Traditional polyp segmentation methods often rely on manually extracted features, such as geometric and texture features [8], shape context information [9], and so on. The limited representational ability of manual features leads to poor segmentation accuracy and fails to adapt to the complex variations in polyps.

Recently, the evolution of deep learning models, especially Convolutional Neural Networks (CNN) [10] and Transformer [11] architectures, has achieved significant breakthroughs in image segmentation, notably improving segmentation precision. Fully Convolutional Networks (FCN) [12] were the first to enable end-to-end training for semantic segmentation, markedly enhancing segmentation accuracy over traditional methods. Following that, Ronneberger et al. [13] developed the well-known U-Net, based on an encoder–decoder structure, innovatively introducing skip connections to enhance the preservation and utilization of feature information at various levels. In 2020, PraNet [14] proposed a parallel partial decoder to amalgamate high-level features, coupled with a reverse attention module to refine polyp boundary details. In 2021, MSNet [15] introduced a subtraction unit to effectively reduce redundant information when merging features from different levels. In 2022, due to the high computational cost of Transformer architectures, Polyp-Mixer [16] utilized an MLP-based encoder–decoder architecture to model long-range dependencies, yet still struggled to meet the real-time requirements of clinical applications. Following that, PolypSeg+ [17] introduced a lightweight context-aware network adaptable to polyp morphological variations, balancing real-time performance with segmentation accuracy, though still falling short of clinical requirements. Overall, models based on CNN struggle to model long-range dependencies, making it difficult to adapt to the large-scale variations of polyps. Models based on the Transformer architecture often find it difficult to maintain real-time performance, thus meeting the demands of clinical applications. Additionally, existing models still cannot adequately balance segmentation efficiency and accuracy.

In this paper, we introduce a novel lightweight long-range context fusion network, designed to address the challenges of real-time polyp segmentation by modeling long-range spatial dependencies without compromising real-time capabilities, thereby better balancing the network’s segmentation accuracy and efficiency. To summarize, the primary contributions of this paper are as follows:

- A novel and efficient lightweight long-range context fusion network, named LightCF-Net, has been proposed for real-time polyp segmentation.
- A novel FAEncoder module has been designed, integrating Large Kernel Attention (LKA) with channel attention mechanisms to extract deep representational features of polyps and discover long-range relationships.
- A novel VAM module has been designed within the skip connections to model long-range contextual dependencies from features extracted by the encoder and to reduce background noise interference by focusing on key information through the attention mechanism.
- Extensive evaluation of the proposed method on four renowned polyp segmentation datasets showed that it surpasses the most advanced lightweight polyp segmentation networks in terms of operational efficiency and segmentation accuracy.

The organization of this article is as follows: Section 2 discusses related work, Section 3 covers the datasets and methods proposed, and Section 4 details the experimental comparison results and analysis. Lastly, the discussion and conclusions are presented in Section 5 and Section 6, respectively.

## 2. Related Work

The widespread application of deep learning technologies has significantly improved the accuracy of polyp segmentation [18]. Akbari et al. [19] used FCN to segment polyps, achieving superior performance over traditional segmentation techniques. Subsequently, the U-shaped network based on encoder–decoder architecture has been widely used. ACSNet [20] first integrates local and global context information to infer polyps of various sizes and shapes. A-DenseUNet [21] combines the advantages of UNet++ and DenseNets, extracting rich multi-scale information between different level encoders. ABC-Net [22] uses two mutually constrained parallel decoders to simultaneously learn and segment polyp regions and boundaries. AG-CUResNeSt [23] introduces the ResNeSt backbone network and attention gates in coupled UNets. SNN [24] simplified the decoder architecture of UNet and introduced the attention mechanism, further improving segmentation accuracy and real-time performance. DCRNet [25] extends beyond capturing context from single images, innovatively exploring inter-image context information. MobileRaNet [26] improved MobileNetV3 using a coordinated attention module, creating the CaNet backbone network with fewer parameters. MFRANet [27] developed an innovative multi-scale feature retention module that effectively preserves base-level spatial features and integrates them with deeper layers, significantly improving segmentation accuracy. SCR-Net [28] introduced semantic calibration and refinement modules to narrow the semantic gap during the fusion of multi-scale features, refining the integrated context information. BUNet [29] focuses on the uncertain areas of colonoscopy images, using a novel boundary exploration module to gradually refine the fine boundaries of polyp regions. sECANet [30] uses the obtained cross-channel interaction information to calibrate channel attention. Chen et al. [31] introduced the self-attention mechanism into the Faster R-CNN architecture, greatly improving the accuracy of polyp detection. Despite the significant advances made by CNN-based approaches, the inherent limitations of convolution operations hinder their ability to model long-range dependencies at higher resolutions, limiting adaptability to large variations in polyp shapes and sizes.

Initially designed for natural language processing, the Transformer model has been successfully adapted for computer vision. Its self-attention mechanism, not confined to a fixed receptive field, enables it to capture long-range spatial dependencies more effectively, leading to its wide application. Numerous studies are aimed at effectively integrating CNNs and Transformers, utilizing the unique strengths of each. TransUNet [32] employs Transformers to re-encode features extracted by CNNs, enhancing its capability for global context understanding. SwinE-Net [33] uses a dual-branch parallel encoder based on CNN’s EfficientNet and ViT’s Swin Transformer to extract rich semantic features, and designs three modules to aggregate and refine the extracted multi-layer features. Polyp-PVT [34] utilizes a pyramid vision Transformer [35] as the encoder to extract multi-scale distant dependency features and proposes three modules for calibrating polyp areas. DS-TransUNet [36] employs a dual-scale encoder made up of two swin transformers to extract features at varying granularities. Nachmani et al. [37] proposed the ResPVT architecture based on Pyramid Vision Transformers and residual blocks, achieving good segmentation performance. However, due to the large number of parameters and computational load of Transformer architectures, it is difficult to maintain real-time performance, failing to meet the needs of clinical applications, prompting researchers to explore methods beyond Transformers for modeling long-range spatial dependencies. Polyp-Mixer [16] uses an MLP-based encoder–decoder architecture to model distant dependencies, but it still struggles to meet the real-time requirements of clinical applications. Recently, state–space models represented by Mamba, due to their lighter computational load and excellent long-range spatial modeling capabilities, have become competitive alternatives to CNN and Transformer architectures. U-Mamba [38] has designed a hybrid encoder that combines CNNs and state–space models, reducing computational load compared to Transformers while capturing long-range dependencies. LightM-UNet [39] employs Mamba blocks in place of the encoder–decoder blocks in U-Nets, also yielding high segmentation accuracy. Although these models have achieved good results, they have not been applied to the field of polyp segmentation. There is still a need to focus on effectively combining local and long-range context information to further enhance polyp segmentation performance and efficiency.

## 3. Materials and Methods

### 3.1. Datasets

To thoroughly evaluate the LightCF-Net proposed in this paper, we conducted extensive experiments on four well-known polyp segmentation datasets: Kvasir-SEG [40], CVC-ClinicDB [41], BKAI-IGH [42], and ETIS [43]. The Kvasir-SEG dataset is currently the largest in the polyp segmentation field, containing 1000 colonoscopy polyp images with various lesion types and image qualities, and provides pixel-level annotations. The CVC-ClinicDB dataset includes 612 polyp images from 31 colonoscopy sequences, with a resolution of 384 × 288 for each image. The BKAI-IGH dataset contains 1000 polyp images with detailed annotation information, with polyps classified into tumorous and non-tumorous types. The ETIS dataset includes 196 annotated polyp images, all with a resolution of 1225 × 966. In the experiments, we employed the widely used dataset setting as used by Nguyen et al. [44], where the dataset images were resized to 320 × 320 and then randomly cropped to 256 × 256 as input to prevent overfitting, with a batch size set to 8. For the validation and test sets, we set the image sizes for the Kvasir-SEG and ETIS datasets to 320 × 320, for the CVC-ClinicDB dataset to 288 × 384, and for the BKAI-IGH dataset to 480 × 480. To comprehensively validate the model performance, we randomly split the datasets into training, validation, and test sets in an 8:1:1 ratio. Furthermore, for a fair comparison, we adopted the aforementioned dataset configuration during training to thoroughly evaluate the segmentation capabilities of all models.

### 3.2. Overall Structure

**Figure 1:**
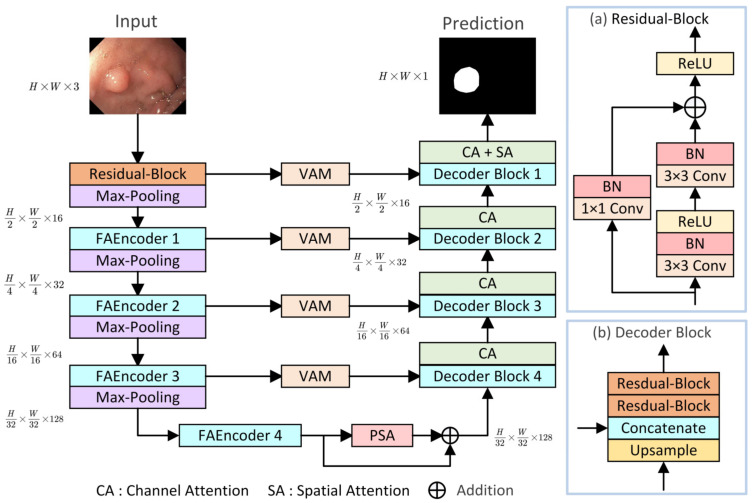
The proposed LightCF-Net network.

The overall structure of the proposed LightCF-Net is illustrated in Figure 1, built upon a U-shaped architecture consisting of encoder–decoder components, mainly comprising three key modules: (1) FAEncoder; (2) VAM; (3) PSA. The encoder of LightCF-Net consists of five stages, with the number of channels per stage being {16, 32, 64, 128, 128}. In the initial stage, to avoid losing excessive image detail and texture information, and to retain more detailed information when fusing features with the last layer decoder, we start with a residual block of kernel size 3. Following this are four stages where the proposed FAEncoder is used to extract deep representational features of polyps. By integrating Large Kernel Attention (LKA) with channel attention mechanisms, the FAEncoder captures long-range correlations, adapting to polyps of various sizes, and further refines polyp discriminative features at the channel level. To further distinguish polyps from the surrounding mucosal tissue, we incorporated the VAM module into the skip connections, which models long-range contextual dependencies with encoder-extracted features and focuses on key information through the attention mechanism to reduce background noise interference. The PSA module is introduced in the bottleneck layer to fully leverage the high-level features extracted by the encoder, capturing a broader range of multi-scale contextual information. Figure 1 illustrates the Decoder Block, with the remaining key components to be detailed in later sections.

### 3.3. Fusion Attention Encoder (FAEncoder)

**Figure 2:**
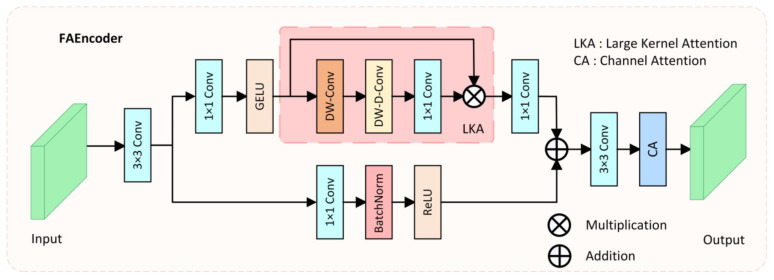
The structure of the proposed Fusion Attention Encoder (FAEncoder).

Due to complex environmental factors in colonoscopy videos, such as insufficient lighting, mucus, or foam residues, it is difficult for the network to effectively extract discriminative features of polyps. Moreover, due to the varying size, color, and morphology of polyps, traditional convolution operations, which only capture features within a small fixed receptive field, make it challenging for the network to fully understand the diverse features of polyps and complex environmental interferences. To address these issues and enhance the encoder’s capacity for diverse feature extraction, we proposed the FAEncoder module. A schematic of the FAEncoder module is depicted in Figure 2.

The FAEncoder module is based on Large Kernel Attention (LKA) [45] and Channel Attention (CA) [46] mechanisms, merging them to produce features with enhanced representational capacity. Generally, larger convolution kernels have a larger receptive field, thus capturing longer dependency relationships. However, large-kernel convolution incurs significant computational costs. The LKA module mitigates this by decomposing the large-kernel convolution into three parts: Depth-wise Convolution (DW-Conv), Depth-wise Dilation Convolution (DW-D-Conv), and Pointwise Convolution (1 × 1 Conv), thus capturing long-range relations and significantly reducing computational complexity. The LKA module first generates attention maps through three distinct convolution operations, then multiplies them with the original input X to produce output features that have captured long-range correlations. The LKA module can be represented as:

(1) $$Attention={Conv}_{1\times 1}(DW\text{-}D\text{-}Conv(DW\text{-}Conv(X))$$

and

(2) LKA=Attention⊗X,

where $X\in R^{C\times H\times W}$ represents the input features, $Attention\in R^{C\times H\times W}$ denotes the attention map, with the values in the attention map signifying the significance of each feature. ⊗ indicates element-wise multiplication.

In the FAEncoder module, for a given input $X_{1}\in R^{C_{1}\times H\times W}$, it first goes through a 3 × 3 convolution, resulting in the feature map $X_{2}\in R^{C_{2}\times H\times W}$. Then, in one branch, it passes through the large kernel attention module to capture long-range correlations, resulting in the output feature $Y_{1}\in R^{C_{2}\times H\times W}$. In another branch, it goes through a 1 × 1 convolution to preserve the original feature information, resulting in the output feature $Y_{2}\in R^{C_{2}\times H\times W}$. The outputs from both branches are added, and following attention adjustment on the channel dimension, the final output $Y_{out}\in R^{C_{2}\times H\times W}$. The computational formula for the FAEncoder module is as follows:

(3) $$Y_{1}={Conv}_{1\times 1}(LKA(GELU({Conv}_{1\times 1}(X_{2}))))$$

and

(4) $$Y_{out}=CA({Conv}_{3\times 3}(Y_{1}⊕Y_{2})),$$

where CA represents the channel attention mechanism, and ⊕ denotes element-wise addition. By adaptively capturing long-range correlations, obtaining a larger receptive field, and optimizing channels, the designed FAEncoder module can capture richer image feature information, facilitating the understanding of diverse polyp features and complex environmental interferences.

### 3.4. Visual Attention Mamba Module (VAM)

**Figure 3:**
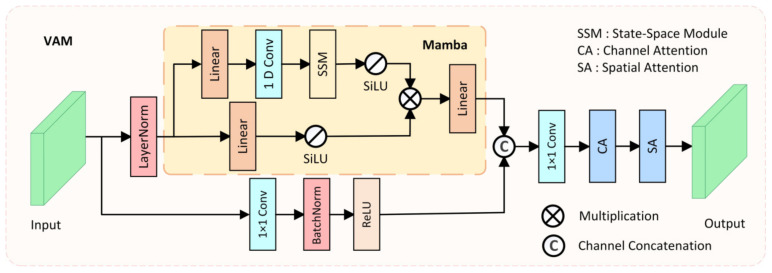
The structure of the proposed Visual Attention Mamba (VAM) module.

Models based on CNNs often struggle to model long-range dependencies due to the fixed receptive field limitation of convolution operations, making it difficult to adapt to large-scale variations in polyps. Models based on Transformer architecture have an advantage in modeling long-range dependencies, which is beneficial for distinguishing polyps from the surrounding ambiguous mucosal tissue. However, the substantial computational cost of the Transformer architecture makes it challenging to meet the real-time requirements of clinical applications. To model long-range spatial dependencies while ensuring real-time performance, we designed the Visual Attention Mamba module (VAM), which incorporates Mamba blocks [47] for long-range spatial modeling, as shown in Figure 3.

In the VAM module, the feature $X_{i}\in R^{C\times H\times W}$ extracted from the i-th layer of the encoder is taken as input and transmitted to two parallel branches. In the first branch, the input feature $X_{i}$ is first flattened and transposed to $W_{i}\in R^{C\times L}$, where L=H×W, and then $W_{i}$ is fed into the Mamba block after LayerNorm. The Mamba block also consists of two branches, where in each branch, the input feature $W_{i}$ is expanded to λ×C channels through a linear layer, with λ being the channel expansion factor. Then, in the first branch of the Mamba block, 1D convolution, SSM, and SiLU [48] activation function are applied in sequence, producing output $Z_{1}\in R^{\lambda \times C\times L}$. In the second branch of the Mamba block, the SiLU activation function is directly applied, producing output $Z_{2}\in R^{\lambda \times C\times L}$. Finally, the outputs of the two branches are multiplied, and after passing through a linear layer to adjust the channel number back to C, the output feature $W_{out}\in R^{C\times L}$ is generated. The mathematical formula for the Mamba block can be represented as:

(5) $$Z_{1}=LayerNorm(SSM(SiLU(1D\;Conv(Linear(W_{1}))))),$$

(6) $$Z_{2}=SiLU(Linear(W_{i}))$$

and

(7) $$W_{out}=Linear(Z_{1}⊗Z_{2}),$$

where ⊗ denotes element-wise multiplication. Finally, the output $W_{out}$ of the Mamba block is reshaped and transposed to the same shape as the original input $X_{i}$, resulting in $W_{out}^{\prime }\in R^{C\times H\times W}$. In the second branch of the VAM module, a 1 × 1 convolution is used to retain the original features extracted by the encoder, resulting in the feature map $X_{2}\in R^{C\times H\times W}$. Then, it is channel-concatenated with the output of the Mamba block, and after optimization through channel–spatial attention, the final output $P_{out}\in R^{C\times H\times W}$ is produced. The mathematical formula for the VAM module can be represented as:

(8) $$P_{out}=SA(CA\left({Conv}_{1\times 1}(Concat(\left[W_{out}^{\prime }\right.,\left.X_{2}\right]))\right)),$$

where SA represents the spatial attention mechanism, CA represents the channel attention mechanism, and Concat denotes channel concatenation. By ensuring real-time performance while modeling long-range spatial dependencies, and integrating various attention mechanisms, the designed VAM module can better distinguish polyps from the surrounding ambiguous mucosal tissue, fulfilling the real-time requirements of clinical applications.

### 3.5. Pyramid Split Attention Module (PSA)

**Figure 4:**
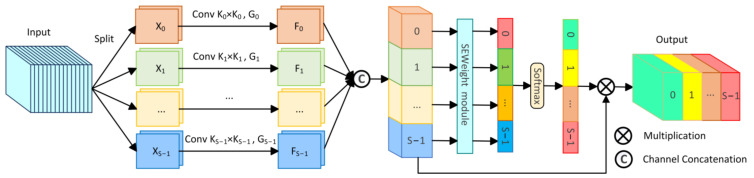
The structure of the Pyramid Split Attention module (PSA).

To comprehensively capture abundant multi-scale information, we incorporated the PSA module [49] into the bottleneck layer of the network. The PSA module, an effective mechanism for multi-scale feature extraction and channel calibration, is depicted in Figure 4.

Initially, the PSA module divides the original input feature $X_{in}\in R^{C\times H\times W}$ into *S* parts through channel division, represented as $\left[X_{0},X_{1},\ldots ,X_{S-1}\right]$, with each part having $\frac{c}{S}$ channels. Then, each section undergoes convolution with kernels of varying sizes to extract multi-scale information. To reduce computational cost, group convolution is introduced and applied in parallel processing of convolution operations, resulting in S feature maps containing information of different scales, represented as $\left[F_{0},F_{1},\ldots ,F_{S-1}\right]$. The entire multi-scale feature extraction operation can be represented as:

(9) $$F_{i}=Conv\left(K_{i}\times K_{i},G_{i}\right)\left(X_{i}\right)\;i=0,1,2\ldots S-1,$$

where $F_{i}\in R^{\frac{C}{S}\times H\times W}$ represents the feature map containing information of different scales, $K_{i}\;$ denotes different convolution kernel sizes, $G_{i}$ represents different group sizes of group convolution, and $K_{i}=2\times \left(i+1\right)+1,\;G_{i}=2^{\frac{K_{i}-1}{2}}$.

Then, the S feature maps containing different scale feature information are concatenated channel-wise to form $F\in R^{C\times H\times W}$. Afterwards, F is applied to the SEweight module and Softmax to recalculate channel weights, obtaining the recalibrated channel weight vector $att=\left[{att}_{0},{att}_{1},\ldots ,{att}_{s-1}\right]$. Finally, the recalibrated weight vector att is multiplied by the feature map $F_{i}$ of the corresponding scale, obtaining the optimized multi-scale feature $T_{out}=\left[T_{0},T_{1},\ldots ,T_{s-1}\right]$, with the calculation formula as follows:

(10) $$F=Concat(\left[F_{0},F_{1},\ldots ,F_{S-1}\right]),$$

(11) $${att}_{i}=\frac{\text{exp}(SEWeight\left(F_{i}\right))}{\sum_{i=0}^{S-1}\text{exp}(SEWeight\left(F_{i}\right))}\;\;i=0,1\ldots S-1,$$

(12) $$att={att}_{0}⊕{att}_{1}⊕···⊕{att}_{S-1}$$

and

(13) $$T_{i}={att}_{i}⊙F_{i}\;\;i=0,1\ldots S-1,$$

where Concat represents channel concatenation, and ⊙ denotes channel-wise multiplication. By incorporating the PSA module in the bottleneck layer, the proposed LightCF-Net can capture richer multi-scale contextual information, thereby better adapting to the irregular variations in the size and shape of tumor polyps.

## 4. Results

### 4.1. Implementation Details

The proposed LightCF-Net was implemented using Pytorch 2.0.0 and Python 3.8 on a computer with an RTX 2080Ti having 11GB of memory. For the experiments, the same learning rate schedule as Zhang et al. [20] was employed, setting the initial learning rate (Init_lr) to 0.001, power to 0.9, maximum training epochs (max_epoch) to 200, and defining the learning rate decay as $\;lr=Init\text{\_}lr\times {\left(1-\frac{epoch}{max\text{\_}epoch}\right)}^{power}$. To enhance data availability and prevent overfitting, we used data augmentation techniques such as horizontal and vertical flips, random cropping, and random rotations between −90 and 90 degrees. Additionally, our model employed an Adam optimizer [50] with a weight decay of 1 × 10^−5^ for parameter optimization, and the batch size was set to 8. Finally, to ensure a fair comparison of all model performances, all experiments were carried out under identical experimental conditions. Additionally, each dataset was partitioned into training, validation, and test sets in an 8:1:1 ratio, and all models followed this same division approach during experimentation, thereby further ensuring consistency in training and testing data across all models.

### 4.2. Loss Function

In order to address the severe class imbalance issue in polyp segmentation tasks, a hybrid loss function, the BCE-Dice Loss, combining Dice loss [51] and binary cross-entropy loss, was employed in the experiments, defined as follows:

(14) $$BCE\text{-}Dice\;Loss=-\sum_{i=1}^{n}\left[G_{i}*\log\left(P_{i}\right)+\left(1-G_{i}\right)*\log\left(1-P_{i}\right)\right]+1-\sum_{i=1}^{n}\frac{2*G_{i}*P_{i}+\sigma }{G_{i}+P_{i}+\sigma },$$

where $G_{i}$ and $P_{i}$ represent the true label and predicted label of the i-th pixel, respectively, and σ denotes a smoothing constant. Additionally, for fair comparison, all comparative networks employ the BCE-Dice loss function.

### 4.3. Comparative Experimental Results and Analysis

To thoroughly evaluate the segmentation performance of the proposed LightCF-Net, we used five evaluation metrics: Intersection over Union (IoU), Dice Similarity Coefficient (DSC), Sensitivity (SE), Specificity (SP), and Accuracy (ACC). Then, we further compared the proposed LightCF-Net against ten advanced models in segmentation efficiency and accuracy, including five large-scale models (U-Net [13], U-Net++ [52], CE-Net [53], DilatedSegNet [54], and Polyp-PVT [34]) and five lightweight models (UNeXt [55], AttaNet [56], LW-IRSTNet [57], DCSAU-Net [58], and PolypSeg+ [17]). Specifically, for a relatively fair comparison, we adopted the same strategy as Wu et al. [17], appropriately reducing the channel numbers of large-scale models to achieve a comparatively smaller size. Secondly, for smaller-sized LW-IRSTNet, we increase their channel numbers to match ours, thus preventing unfairness in comparative experiments due to their smaller sizes. Within an identical computing environment, we applied the same data partitioning and augmentation methods to implement ten competing models on four datasets. All models were retrained with the same iteration count, batch size, loss function, and optimizer, without any pre-trained weights, which maximally reduced training discrepancies. The comparison results on the Kvasir-SEG and CVC-ClinicDB datasets are presented in Table 1.

The experimental results in Table 1 show that our proposed method surpasses other methods on the Kvasir-SEG and CVC-ClinicDB datasets in terms of evaluation metrics. Specifically, on the Kvasir-SEG dataset compared to large-scale models, our method leads the second-place Polyp-PVT by 0.90% in IoU and 0.57% in DSC, and is only slightly behind DilatedSegNet by 0.53% in SE. On the Kvasir-SEG dataset, against lightweight models, our method is ahead of the second-placed PolypSeg+ by 1.51% in IoU and 1.02% in DSC. Against large-scale models on the CVC-ClinicDB dataset, our method surpasses the second-ranking Polyp-PVT by 0.82% in IoU and 0.47% in DSC. On the CVC-ClinicDB dataset, in comparison with lightweight models, our method outperforms the second-best PolypSeg+ by 1.82% in IoU and 1.04% in DSC. The comparison results on the BKAI-IGH and ETIS datasets are presented in Table 2.

The experimental results in Table 2 show that our proposed method surpasses other methods on the BKAI-IGH and ETIS datasets in terms of evaluation metrics. Specifically, on the BKAI-IGH dataset compared to large-scale models, our method leads the second-place Polyp-PVT by 3.24% in IoU and 2.06% in DSC, and is only slightly behind U-Net++ by 0.11% in SE. On the BKAI-IGH dataset, against lightweight models, our method is ahead of the second-placed PolypSeg+ by 1.83% in IoU and 1.14% in DSC. Against large-scale models on the ETIS dataset, our method surpasses the second-ranking Polyp-PVT by 1.26% in IoU and 0.9% in DSC. On the ETIS dataset, in comparison with lightweight models, our method outperforms the second-best PolypSeg+ by 2.34% in IoU and 1.74% in DSC. The experimental findings reveal that our method exhibits superior segmentation performance compared to other methods, particularly when compared to other lightweight models. Moreover, this also shows that the proposed lightweight feature extraction and optimization modules can effectively address the main challenges in polyp segmentation tasks.

**Figure 5:**
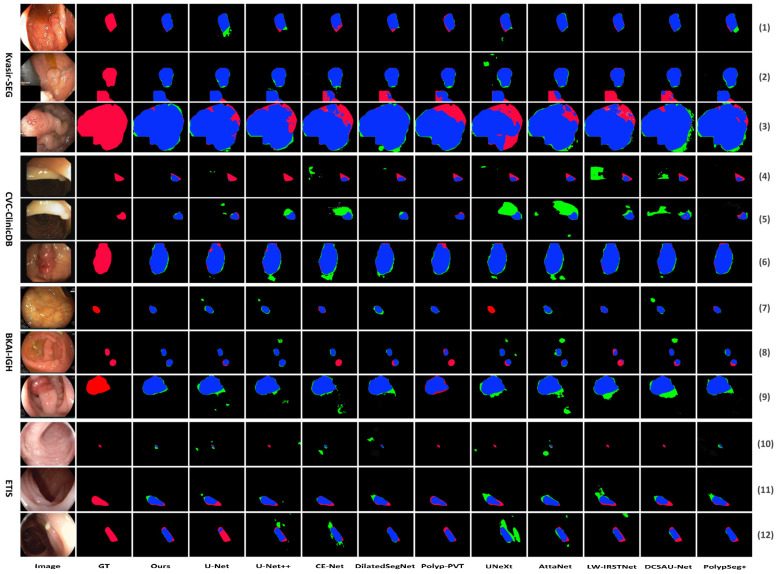
Visual comparison of segmentation performance of the compared networks (TP and FP pixels are represented in blue and green, respectively, while FN pixels are represented in red).

The visual comparison results are depicted in Figure 5. Figure 5 shows the segmentation comparison results of the proposed LightCF-Net and other comparative models on some challenging polyp examples, including polyps with varying shapes, sizes, colors, and those with low contrast against the surrounding mucosal tissue. Figure 5 reveals that, with a relatively fixed receptive field, other networks have difficulty adapting to complex environmental disruptions, as shown in the 4th row with darker lighting and the 5th and 12th rows with stronger lighting in Figure 5. Furthermore, compared to other models, LightCF-Net can capture longer-range spatial dependency information and more accurately distinguish between polyps and unclear mucosal tissues, as shown in rows 3, 9, and 11 of Figure 5. In addition, LightCF-Net also has advantages in identifying small polyps, as shown in rows 7, 8, and 10 of Figure 5. On some relatively easier-to-distinguish polyp examples, LightCF-Net also performs well, producing smoother and more accurate edge contours that are closer to the true boundaries, as illustrated in the first, second, and sixth rows of Figure 5.

More importantly, we calculated the parameters, FLOPs, and FPS for each method on the common NVIDA GeForce 3060 Laptop GPU and CVC-ClinicDB dataset to assess their segmentation efficiency, as shown in Table 3. As seen in Table 3, our method is second only to AttaNet and U-Net, outperforming other lightweight models, indicating that our method can produce better segmentation results in less time, which is more beneficial for clinical applications.

**Figure 6:**
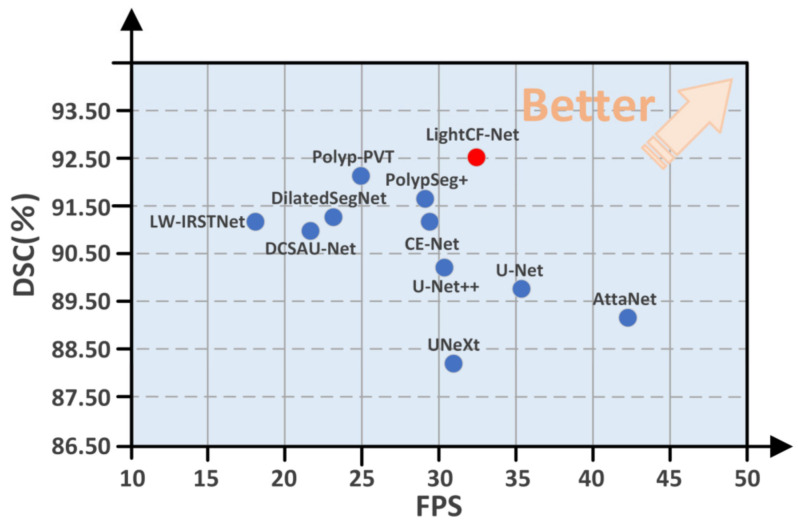
Comparative performance of the networks in DSC and FPS metrics. The proposed LightCF-Net network is represented by a red dot. The closer to the direction of the arrow, the better the performance of the model.

Moreover, to highlight the superiority of the proposed model, we compared the DSC and FPS metrics of all models on the CVC-ClinicDB test set, as illustrated in Figure 6. As evident from Figure 6, our proposed LightCF-Net achieves a better balance between segmentation efficiency and precision.

**Figure 7:**
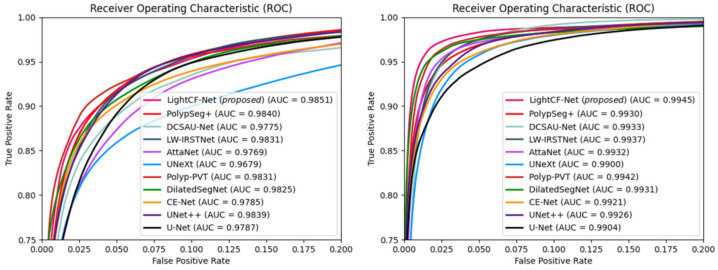
The ROC curves and AUC values for the networks compared on the Kvasir-SEG dataset (**left**) and CVC-ClinicDB dataset (**right**).

Additionally, we calculated the Area Under the ROC Curve (AUC) and Receiver Operating Characteristic (ROC) values for different models on the Kvasir-SEG and CVC-ClinicDB datasets to further assess the segmentation performance of each model, as illustrated in Figure 7. Figure 7 shows that our model possesses the highest AUC values on both datasets, and its ROC curve is nearest to the top-left corner, signifying the superior overall accuracy of LightCF-Net.

### 4.4. Ablation Study

To evaluate the effectiveness of modules in the proposed LightCF-Net, we conducted ablation studies on the Kvasir-SEG dataset, with the results shown in Table 4. The channel count for each layer of the U-Net was configured as {16, 32, 64, 128, 128}, establishing it as the baseline network.

From Table 4, it can be seen that the baseline network had IoU and DSC values of 74.49% and 85.38%, respectively. The inclusion of the FAEncoder module without channel attention mechanism resulted in increases of 1.12% in IoU and 0.73% in DSC. With the addition of the complete FAEncoder module, IoU and DSC values increased by 2.16% and 1.4%, respectively. Adding the VAM module led to increases of 2.18% in IoU and 1.42% in DSC, respectively. When both FAEncoder and VAM modules were added, IoU and DSC values increased by 3.34% and 2.15%, respectively.

**Figure 8:**
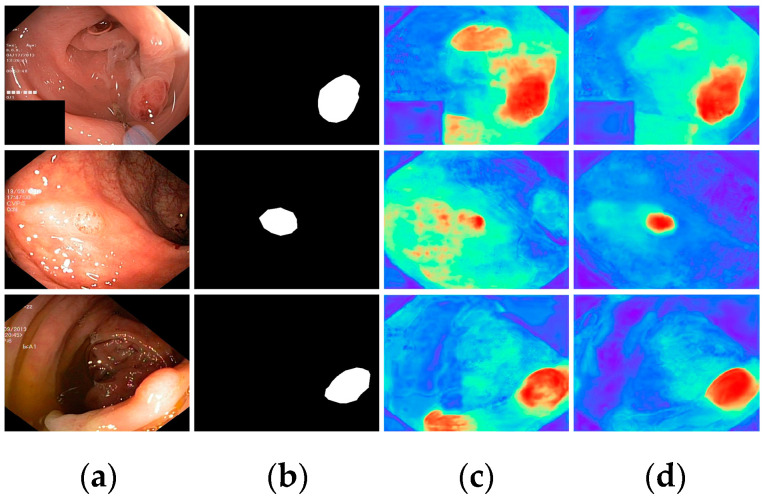
Visual comparison of the ablation study for the FAEncoder module: (**a**) represents the original image, (**b**) the ground truth, (**c**) the heatmap extracted by the baseline network, and (**d**) the heatmap extracted by the baseline network with FAEncoder. Red indicates that the network is paying more attention to the area, while blue indicates that the network is paying less attention to the area. Brighter colors indicate greater levels of concern.

**Figure 9:**
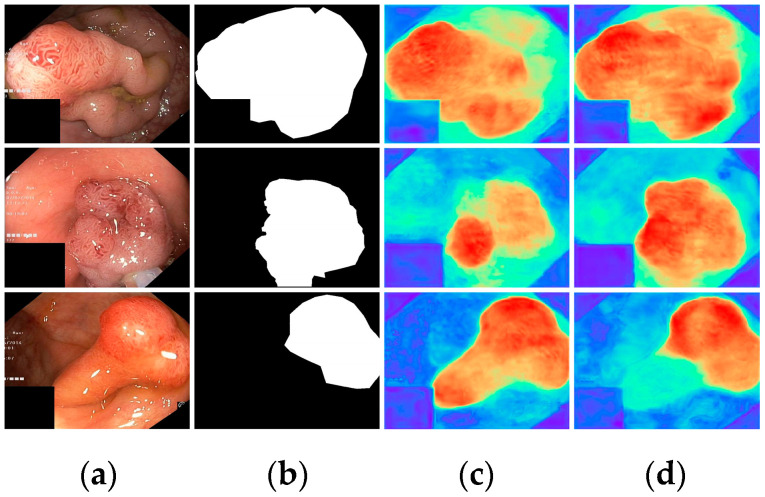
Visual comparison of the ablation study for the VAM module: (**a**) represents the original image, (**b**) the ground truth, (**c**) the heatmap extracted by the baseline network, and (**d**) the heatmap extracted by the baseline network with VAM. Red indicates that the network is paying more attention to the area, while blue indicates that the network is paying less attention to the area. Brighter colors indicate greater levels of concern.

Finally, with all modules incorporated, all metrics demonstrated optimal results, highlighting the effectiveness of the proposed modules. To further visualize the effectiveness of the different modules, we displayed the attention heatmaps of the baseline network with added FAEncoder and VAM modules in Figure 8 and Figure 9, respectively. Figure 8 reveals that with the FAEncoder module added, the network can concentrate more on the polyp area, minimizing background noise interference. Figure 9 shows that with the incorporation of the VAM module, the network can more precisely differentiate between polyps and unclear mucosal tissues. The above experiments fully demonstrate the effectiveness of the extracted modules.

## 5. Discussion

Based on the comparative results from Table 1 and Figure 5, the proposed LightCF-Net model performs well in addressing the challenges of polyp segmentation, adapting effectively to tumors of various sizes and shapes, yet the method still has some limitations. Firstly, although our method achieves good segmentation accuracy, the complexity of the clinical environment and the limitations of the labeled data during training mean that it still struggles to perfectly meet the challenges of clinical applications; in the future, we will consider semi-supervised and unsupervised networks. Secondly, the real-time performance of the proposed method still requires further enhancement. Improving the network’s generalization capabilities and real-time performance will be the focus of our future research.

## 6. Conclusions

Complex environmental factors in colonoscopy videos, such as insufficient lighting, mucus, or foam residues, make it difficult for existing networks to effectively extract discriminative features of polyps. Furthermore, due to the varying size, color, and shape of polyps, developing a segmentation network that is suitable for all types of polyps and consistently achieves satisfactory performance is very challenging. Moreover, in clinical settings, segmentation algorithms need to have high real-time capability so that physicians can promptly diagnose and treat. To address these issues, this paper proposes a novel lightweight long-range context fusion network for real-time polyp segmentation, named LightCF-Net. Initially, the LightCF-Net uses the FAEncoder, which integrates Large Kernel Attention (LKA) and channel attention mechanisms, to extract deep semantic features and discover long-range correlations. Secondly, a newly designed Visual Attention Mamba module (VAM) is incorporated into the network’s skip connections, modeling long-range contextual dependencies in the features extracted by the encoder and focusing on key information through the attention mechanism to reduce background noise. Lastly, a Pyramid Split Attention module (PSA) is added to the bottleneck layer of the network, to capture richer multi-scale contextual feature information. Extensive experiments on the renowned polyp segmentation datasets Kvasir-SEG and CVC-ClinicDB have demonstrated that LightCF-Net achieves higher segmentation accuracy in less time compared to other state-of-the-art networks, making it more suitable for clinical applications.

## Figures and Tables

**Table 1:** Comparison results on the Kvasir-SEG and CVC-ClinicDB datasets, with the best outcomes in bold.

Method	Kvasir-SEG					CVC-ClinicDB				
	IoU (%)	DSC (%)	SE (%)	SP (%)	ACC (%)	IoU (%)	DSC (%)	SE (%)	SP (%)	ACC (%)
U-Net	75.98	86.35	85.01	97.49	95.32	81.33	89.70	87.82	99.06	98.28
U-Net++	76.10	86.43	85.11	97.50	95.34	82.47	90.39	88.56	98.94	98.39
CE-Net	76.74	86.84	84.30	97.92	95.54	83.89	91.24	93.69	98.91	98.46
DilatedSegNet	77.16	87.11	**85.36**	97.48	95.54	84.16	91.40	93.15	99.00	98.50
Polyp-PVT	77.75	87.48	82.79	98.23	95.87	85.27	92.05	88.48	99.05	98.69
UNeXt	72.42	84.00	80.04	97.78	94.69	78.87	88.19	88.99	98.80	97.96
AttaNet	72.51	84.07	81.43	97.40	94.62	80.48	89.19	91.76	98.69	98.10
LW-IRSTNet	76.25	86.52	82.76	98.20	95.51	83.95	91.27	92.97	99.00	98.48
DCSAU-Net	76.74	86.84	83.37	98.17	95.59	83.80	91.19	90.67	99.13	98.50
PolypSeg+	77.14	87.03	83.28	98.32	95.70	84.27	91.48	90.95	99.04	98.55
Ours	**78.65**	**88.05**	84.83	**98.34**	**95.99**	**86.09**	**92.52**	**93.81**	**99.16**	**98.70**

**Table 2:** Comparison results on the BKAI-IGH and ETIS datasets, with the best outcomes in bold.

Method	BKAI-IGH					ETIS				
	IoU (%)	DSC (%)	SE (%)	SP (%)	ACC (%)	IoU (%)	DSC (%)	SE (%)	SP (%)	ACC (%)
U-Net	73.52	84.74	82.30	99.40	98.58	61.35	76.04	67.17	99.14	98.87
U-Net++	74.62	85.47	**85.90**	99.17	98.58	63.90	77.97	77.25	99.03	98.84
CE-Net	74.89	85.64	82.11	99.51	98.67	64.28	78.25	79.05	99.17	98.83
DilatedSegNet	75.12	85.79	83.53	99.44	98.67	65.47	78.96	86.67	99.13	98.82
Polyp-PVT	75.78	86.22	83.09	99.45	98.80	66.74	80.05	86.15	99.19	98.89
UNeXt	66.19	79.66	78.52	99.06	98.08	56.33	72.06	76.33	99.03	98.42
AttaNet	70.06	82.40	82.77	99.09	98.30	60.48	75.37	**87.63**	98.77	98.47
LW-IRSTNet	73.65	84.83	84.16	99.37	98.58	63.78	77.88	86.37	99.01	98.68
DCSAU-Net	74.70	85.52	85.75	99.25	98.61	64.81	78.64	76.34	99.16	98.89
PolypSeg+	77.19	87.14	85.26	99.28	98.61	65.66	79.21	87.09	99.18	98.85
Ours	**79.02**	**88.28**	85.79	**99.57**	**98.91**	**68.00**	**80.95**	86.41	**99.26**	**98.92**

**Table 3:** Comparison of segmentation efficiency among various advanced models, with the best results shown in bold.

Method	Params (M)	FLOPs (G)	FPS
U-Net [13]	7.24	12.16	36
U-Net++ [52]	9.16	34.7	31
CE-Net [53]	3.12	3.32	29
DilatedSegNet [54]	2.88	10.14	23
Polyp-PVT [34]	3.65	1.79	25
UNeXt [55]	1.47	**0.57**	32
AttaNet [56]	**1**	1.64	**43**
LW-IRSTNet [57]	2.48	8.91	22
DCSAU-Net [58]	2.59	13.83	18
PolypSeg+ [17]	2.54	7.23	28
LightCF-Net (Ours)	1.52	3.25	33

**Table 4:** Ablation study results on the Kvasir-SEG dataset, with the best results in bold.

Method	Params (M)	FLOPs (G)	IoU (%)	DSC (%)	SE (%)	SP (%)	ACC (%)
Baseline	0.97	2.67	74.49	85.38	82.29	97.79	95.09
Baseline + FAEncoder_w/o_CA	1.15	2.97	75.61	86.11	82.87	97.97	95.34
Baseline + FAEncoder	1.16	2.97	76.65	86.78	83.83	98.02	95.55
Baseline + VAM	1.21	2.91	76.67	86.80	84.28	97.63	95.48
Baseline + FAEncoder + VAM	1.40	3.22	77.83	87.53	84.60	98.16	95.80
Baseline (PSA) + FAEncoder + VAM	1.52	3.25	**78.65**	**88.05**	**84.83**	**98.34**	**95.99**

## Data Availability

The Kvasir-SEG dataset used in the experiments is available at https://datasets.simula.no/kvasir-seg/ (accessed on 23 April 2024), and the CVC-ClinicDB dataset can be found at https://polyp.grand-challenge.org/CVCClinicDB/ (accessed on 23 April 2024).
